# *Arcobacter cryaerophilus* Isolated From New Zealand Mussels Harbor a Putative Virulence Plasmid

**DOI:** 10.3389/fmicb.2019.01802

**Published:** 2019-08-05

**Authors:** Stephen L. W. On, Damien Althaus, William G. Miller, Darrell Lizamore, Samuel G. L. Wong, Anso J. Mathai, Venkata Chelikani, Glen P. Carter

**Affiliations:** ^1^Department of Wine, Food and Molecular Biosciences, Lincoln University, Lincoln, New Zealand; ^2^Produce Safety and Microbiology Research Unit, Agricultural Research Service, U.S. Department of Agriculture, Albany, CA, United States; ^3^Doherty Applied Microbial Genomics, The Peter Doherty Institute for Infection and Immunity, University of Melbourne, Melbourne, VIC, Australia

**Keywords:** *Arcobacter cryaerophilus*, shellfish, mussel, pathogen, virulence plasmid

## Abstract

A wide range of *Arcobacter* species have been described from shellfish in various countries but their presence has not been investigated in Australasia, in which shellfish are a popular delicacy. Since several arcobacters are considered to be emerging pathogens, we undertook a small study to evaluate their presence in several different shellfish, including greenshell mussels, oysters, and abalone (paua) in New Zealand. *Arcobacter cryaerophilus*, a species associated with human gastroenteritis, was the only species isolated, from greenshell mussels. Whole-genome sequencing revealed a range of genomic traits in these strains that were known or associated virulence factors. Furthermore, we describe the first putative virulence plasmid in *Arcobacter*, containing lytic, immunoavoidance, adhesion, antibiotic resistance, and gene transfer traits, among others. Complete genome sequence determination using a combination of long- and short-read genome sequencing strategies, was needed to identify the plasmid, clearly identifying its benefits. The potential for plasmids to disseminate virulence traits among *Arcobacter* and other species warrants further consideration by researchers interested in the risks to public health from these organisms.

## Introduction

The genus *Arcobacter* currently contains 26 species (Pérez-Cataluña et al., 2018) of diverse origin, from cases of human diarrhea, and from livestock and aquatic environments, including shellfish (Ferreira et al., 2016; Ramees et al., 2017; Pérez-Cataluña et al., 2018). Indeed, in recent years, many new *Arcobacter* species have been recovered from shellfish, including *Arcobacter bivalviorum* (Levican et al., 2012), *Arcobacter canalis* (Pérez-Cataluña et al., 2018), *Arcobacter molluscorum* (Figueras et al., 2011a), *Arcobacter ellisii* (Figueras et al., 2011b), *Arcobacter mytili* (Collado et al., 2009) and *Arcobacter venerupis* (Levican et al., 2012). The relatively recent description of these species makes an evaluation of their potential threat to human health, or pathogenic potential, problematic. However, other species, including *A. butzleri*, *Arcobacter cryaerophilus*, and *Arcobacter skirrowii*, were among the first to be classified into the genus in the early 1990s (Vandamme et al., 1991, 1992) and are considered emerging pathogens warranting further study (International Commission on Microbiological Specifications for Foods [ICMSF], 2002; Ferreira et al., 2016; Ramees et al., 2017). Several studies have demonstrated the presence of these species in shellfish, in some cases in 100% of the samples examined (reviewed by Hsu and Lee, 2015).

In New Zealand, shellfish are an important component of the diet of, notably, indigenous (Mâori) New Zealanders (Ministry of Health, 2012). Shellfish can be eaten raw and so pose a special risk to consumers from a food safety perspective. Although the risks to human health from more established seafood pathogens such as *Vibrio* species have been investigated in New Zealand (Cruz et al., 2015, 2016), no study to our knowledge has previously investigated shellfish of Australasian origin for *Arcobacter* species. Nonetheless, emerging pathogenic *Arcobacter* species have been detected in various production- and domestic animals in New Zealand (McFadden et al., 2005; Bojanić et al., 2017, 2019).

We report here results from a small study in which locally sourced shellfish were examined for those *Arcobacter* species implicated as emerging pathogens, and isolates subjected to phenotypic and genotypic testing, including whole-genome sequencing (WGS).

## Materials and Methods

### Isolation and Phenotypic Characterization of *Arcobacter* spp. From New Zealand Shellfish

Recovery of *Arcobacter* spp. was attempted from greenshell mussels (five batches from two regions in the South Island of 8–20 animals each), oysters (one batch from the Bluff region, *n* = 12), and abalone (Paua, received frozen, exact place of origin unknown, one batch, *n* = 10). Shellfish were harvested between 7.3.2016 and 23.5.2016 1 day prior to examination, using methods described previously (Levican et al., 2014) with minor modifications. Eight grams of shellfish meat were incubated overnight at room temperature (18–22°C) in 80 ml of Cefoperazone Amphotericin Teicoplanin (Oxoid Ltd., Basingstoke, United Kingdom) broth contained in 100 ml Schott bottles. Subsequently, 100 μl aliquots were inoculated onto blood agar plates, and incubated as prescribed (Levican et al., 2014) for up to 7 days at room temperature and 30°C. Suspect colonies underwent phenotypic analyses, including: cell morphology assessment, catalase activity, indoxyl acetate hydrolysis, nitrate reduction, growth at 37°C, and growth on 1% glycine, 4% NaCl-containing media, and *Campylobacter* Blood-Free Selective Agar Base [Oxoid, CM0739]. The colonies were also antibiotyped with standardized methods as recommended (On et al., 1996, 2017). In brief, suspensions of 3-day old bacterial cultures were made in nutrient broth no. 2 (Oxoid Ltd.) of a density equating to ca. 10^6^ colony forming units/ml and seeded onto Mueller-Hinton agar (Oxoid) supplemented with 5% calf blood. Antibiotic disks were placed onto these plates and zones of inhibition determined after 3 days incubation at 30°C in aerobic conditions.

### Whole-Genome Sequencing, Annotation, and Plasmid Screening

Genomic DNA was extracted and sequenced using both short- (NextSeq 500 platform, Illumina, San Diego, CA, United States) and long-read (RS II platform, Pacific Biosciences, Menlo Park, CA, United States) technologies (Miller et al., 2018) for two isolates (M830MA and G13RTA); and the short read platform only for the remaining two strains (M830A and G18RTA), due to financial constraints. Genomes were assembled using SPAdes v3.9 and annotated using automated and manual approaches, as described elsewhere (Seemann, 2014; Miller et al., 2018). Genes with virulence potential were identified by reference to extant Genbank annotations and/or by cross-referencing to peer-reviewed publications. Plasmid carriage was confirmed using a QIAprep Spin Miniprep Kit (Qiagen, Hilden, Germany) with DNA content confirmed by Nanodrop (Thermo Fisher Scientific Ltd., Auckland, New Zealand), using the manufacturers recommendations.

### Phylogenetic and *in silico* DNA–DNA Hybridization Analyses

Housekeeping gene sequences (16s rRNA, *atpA*, *rpoB*, and *groEL*) were extracted and compared with corresponding sequences from validly described *Arcobacter* spp. as described previously (On et al., 2017). *In silico* DNA–DNA hybridizations between our shellfish isolates and those of extant species were undertaken using Genome Blast Distance Phylogeny (GBDP) (Meier-Kolthoff et al., 2014), with parameters recommended for *Arcobacter* and related organisms (On et al., 2017).

## Results

### Isolation, Identification, and Antibiotyping of Strains

Four *Arcobacter* spp. strains were recovered from two of the five batches of greenshell mussels examined, harvested in March (from the Kenepuru Sound growing area) and May 2016 (from the Admiralty Bay growing area), respectively. Arcobacters were not recovered from the other three mussel batches, or the oyster and Paua samples. Three strains were isolated in aerobic conditions and the fourth in microaerobic conditions. The phenotyping undertaken correlated well with corresponding data obtained for *A. cryaerophilus* (On et al., 1996), although nitrate was not reduced. Disk diffusion-based antibiotyping determined complete resistance to nalidixic acid (30 μg) and vancomycin (5 μg), and intermediate resistance to ceftaroline (30 μg), chloramphenicol (30 μg), cefoxitin (30 μg) and tetracycline (30 μg) in all strains.

**FIGURE 1:**
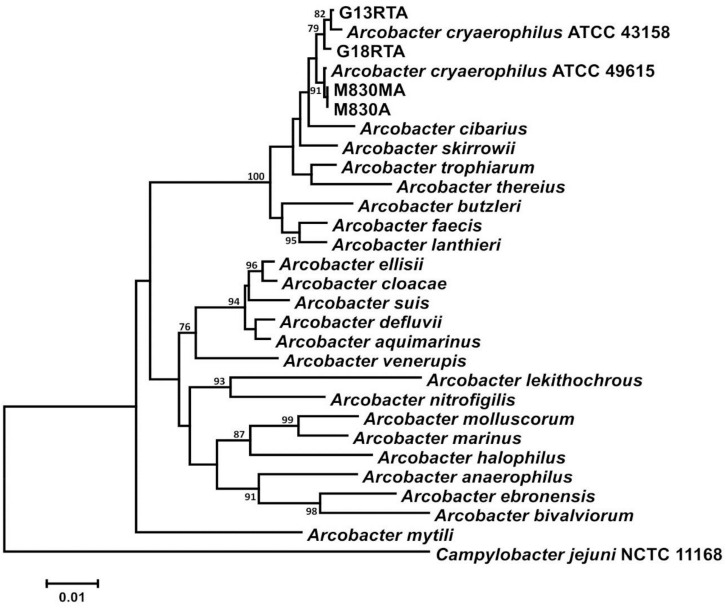
16S rRNA gene analysis of New Zealand *Arcobacter* isolates from mussels with other validly described species showing a clustering with type and reference strains of *Arcobacter cryaerophilus*.

Phylogenetic analysis of each of the housekeeping gene sequences used clustered New Zealand mussel isolates together with type and reference strains of *A. cryaerophilus*. The 16S rRNA gene comparison is presented here as an exemplar (Figure 1). The whole-genome sequences of two isolates [M830A and M830MA (Genbank SNQM01000000 and CP026656, respectively)] from the same batch recovered under aerobic and microaerobic conditions, respectively, possessed identical housekeeping gene sequences, protein profiles, and phenotypes, implying they represent the same clone. The remaining strains [G13 and G18 (Genbank CP026655 and SNQL01000000, respectively)] harbored unique genome sequences. Quantitative DNA–DNA hybridization values, as predicted from GBDP analyses of the whole-genome sequences, showed that the New Zealand mussel strains were 72.7–78.5% similar to those of a well-characterized reference strain (ATCC 49615) of *A. cryaerophilus* subgroup 2 (Vandamme et al., 1992). These values are well within accepted taxonomic boundaries for *Arcobacter* and related species, using the methods described (On et al., 2017). All our taxonomic data identify these strains as *A. cryaerophilus*.

### Genome and Plasmid Analysis

Following Illumina sequencing, approximately 130× to 160× read depth was obtained per isolate and for PacBio sequencing, approximately 115× coverage was obtained. Genome sizes of the four isolates examined were each in the region of 2.1 MB in size. Analysis of the complete genome of M830MA identified a putative virulence plasmid (BankIt2207814 M830MA_plasmid MK715471). Plasmid carriage was confirmed independently in this strain and in M830A (i.e., the clone recovered from the same batch using aerobic conditions) using the Qiaprep kit (data not shown). Bioinformatic analysis of the draft (produced using the short-read sequencing method) genome sequence determined for M830A did not identify a plasmid present. Plasmids were not detected in strains G13 or G18 either with the Qiaprep kit or bioinformatic analysis of the genome sequences.

**FIGURE 2:**
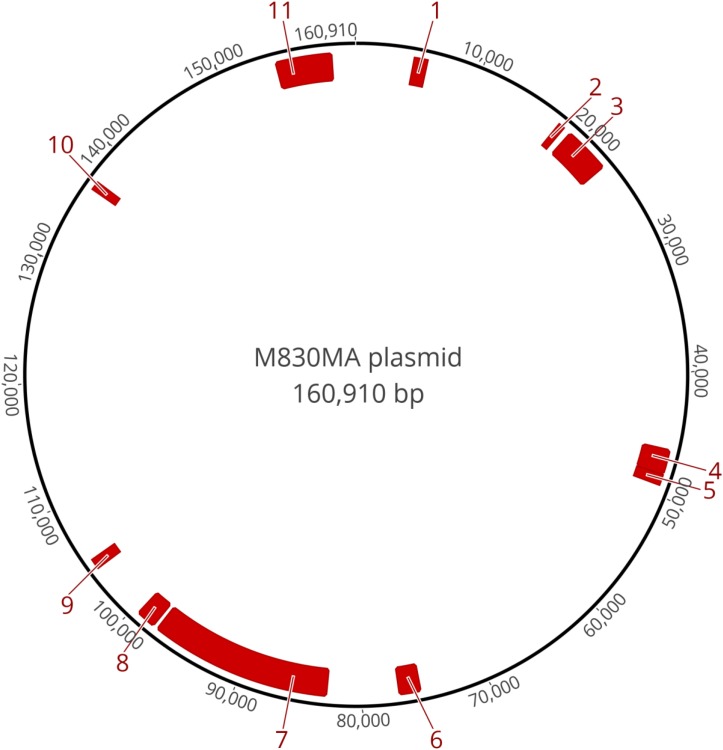
Schematic of the circularized plasmid sequence and position of the gene clusters that are associated with putative virulence traits, as annotated in Table 1.

Annotation of the 160,910 bp plasmid sequence in strain M830MA identified 150 genes, 95 of which were not associated with any known function. Table 1 summarizes the size, location and predicted function of the remaining 55 genes, 15 were known, or associated with, virulence determinants such as adhesion, invasion, immunoavoidance, antimicrobial resistance (AMR), and biofilm formation. Several clusters of these genes are evident (Figure 2).

**TABLE 1:** List of genes identified on the *A. cryaerophilus* virulence plasmid, showing predicted size, location, and function.

**Start location**	**Stop location**	**Product [source if known]**	**Known/potential role in virulence**	**Cluster**	**% Identity to annotated gene**
1	939	WP_105918336.1 integrase [*Arcobacter cryaerophilus*]			100.00%
985	2001	D-alanine-D-alanine ligase			
4663	5805	WP_105918343.1 Fic family protein [*Arcobacter cryaerophilus*]	Leads to cell death (Engel et al., 2012)	1	97.40%
6386	7576	WP_105918342.1 ATP-binding protein [*Arcobacter cryaerophilus*]			100.00%
9061	8501	WP_066151948.1 XRE family transcriptional regulator [*Arcobacter cryaerophilus*]	Plasmid preservation		100.00%
17397	17969	WP_105916127.1 GNAT family N-acetyltransferase [*Arcobacter cryaerophilus*]	Potential involvement with Antimicrobial Resistance (Vetting et al., 2005)	2	85.30%
18532	22383	WP_105917898.1 filamentous hemagglutinin domain-containing protein	Epithelial cell adhesion (Asakura et al., 2012)	3	72.40%
23027	25437	Mobile element, insertion sequence ISM830-1A			
23080	24618	WP_066355114.1 IS21 family transposase [*Arcobacter skirrowii*]			96.30%
25131	25388	WP_066357872.1 transposase [*Arcobacter cryaerophilus*]			100.00%
27550	28437	WP_105916124.1 nucleotidyl transferase AbiEii/AbiGii toxin family protein [*Arcobacter cryaerophilus*]			96.90%
32061	32381	WP_105913889.1 thioredoxin [*Arcobacter cryaerophilus*]			85.80%
37006	35732	WP_090568776.1 DUF4071 domain-containing protein [*Nitrosomonas* sp. Nm33]			49.60%
46206	46003	WP_033698421.1 MULTISPECIES: DUF4062 domain-containing protein [*Pseudomonas*]			47.60%
48260	46218	Patatin-like phospholipase	Invasion/Lipase activity (Anderson et al., 2015)	4	
49149	48313	WP_080353957.1 toll/interleukin-1 receptor domain-containing protein	Immunoavoidance (Ve et al., 2015)	5	37.30%
52050	50725	Replicative DNA helicase			
53555	52065	WP_081754537.1 replication initiation protein [*Arcobacter faecis*]			97.40%
56007	54991	ParB family protein (product partitioning)			
56931	56017	WP_066152783.1 ParA family protein [*Arcobacter cryaerophilus*]			100.00%
58421	57357	NT_Rel-Spo_like domain-containing protein			
58822	60015	Putative exonuclease subunit SbccD, D subunit			86.10%
60012	63590	Putative exonuclease subunit SbccD, C subunit			82.00%
66465	67940	WP_066152788.1 DUF2779 domain-containing protein [*Arcobacter cryaerophilus*]			96.50%
72016	73461	WP_066152765.1 dGTPase [*Arcobacter cryaerophilus*]			100.00%
75193	74501	WP_105918093.1 2- component system response regulator			97.80%
77075	75249	7TMR-DISM-7TM/7TMR-DISMED2 domain-containing signal transduction protein	Carbohydrate binding, possible role in biofilm dispersion (Basu Roy and Sauer, 2014)	6	
82841	92395	WP_066402993.1 RTX toxin-related calcium-binding protein	Cytotoxic activity (Linhartová et al., 2010)	7	90.60%
92408	92848	WP_066152392.1 toxin-activating lysine-acyltransferase [*Arcobacter cryaerophilus*]	Possible hemolysin activator (Greene et al., 2015)	7	100.00%
94123	96261	WP_066152387.1 type I secretion system permease/ATPase [*Arcobacter cryaerophilus*]	Protein export	7	93.00%
96262	97584	WP_066403004.1 HlyD family type I secretion periplasmic adaptor subunit [*Arcobacter cryaerophilus*]	Protein export	7	97.00%
97900	99486	WP_026806319.1 type II toxin-antitoxin system HipA family toxin [*Arcobacter faecis*]	AMR/persister cell formation (Correia et al., 2006)	8	97.70%
103041	100630	Mobile element, insertion sequence ISM830-1B			
101425	100679	Transposase-associate protein, IS21 family			
102973	101450	WP_066355114.1 IS21 family transposase [*Arcobacter skirrowii*]			96.30%
105128	104178	Patatin-like phospholipase	Invasion/Lipase activity (Anderson et al., 2015)	9	67.00%
105545	106747	WP_009379108.1 nucleotidyltransferase [*Bilophila* sp. 4_1_30]			50.50%
107987	107631	Toxin-antitoxin system, antitoxin component, RnlB family			
109038	107974	Toxin-antitoxin system, antitoxin component, RnlA family			
110430	111521	Site-specific recombinase			
119887	120141	WP_105918348.1 XRE family transcriptional regulator [*Arcobacter cryaerophilus*]			97.60%
127306	129717	Mobile element, insertion sequence IS830-1C			
127374	128897	WP_066355114.1 IS21 family transposase [*Arcobacter skirrowii*]			96.30%
128922	129668	WP_046996155.1 MULTISPECIES: transposase [*Arcobacter*]			94.00%
131416	133359	WP_090294727.1 DUF4365 domain-containing protein [*Muricauda zhangzhouensis*]			29.10%
137132	136389	WP_090938743.1 TIR domain-containing protein [*Azotobacter beijerinckii*]	Immunoavoidance (Ve et al., 2015)	10	70.40%
138225	137221	WP_015487510.1 DUF4917 domain-containing protein [*Thalassolituus oleivorans*]			82.00%
140934	140371	WP_066152761.1 EamA/RhaT family transporter [*Arcobacter cryaerophilus*]			93.60%
141676	141299	WP_066152763.1 AraC family transcriptional regulator [*Arcobacter cryaerophilus*]			100.00%
142281	142844	WP_066152060.1 recombinase family protein [*Arcobacter cryaerophilus*]			98.90%
148254	150416	Glycosyl hydrolase			
152741	152118	DUF4263 domain-containing protein			
154436	154597	Alpha/beta hydrolase	Invasion/Lipase activity	11	
157284	158967	Patatin-like phospholipase	Invasion/Lipase activity (Anderson et al., 2015)	11	
159486	160694	Site-specific tyrosine recombinase, phage integrase family			

*Virulence gene clusters are labeled 1–12 according to location and function. Pseudogenes and genes coding for hypothetical proteins are not listed.*

## Discussion

Of the *Arcobacter* spp. known, *A. cryaerophilus* is among the most commonly detected (Ferreira et al., 2016), and there have been various reports of *A. cryaerophilus*-associated human gastroenteritis, including in New Zealand (Mandisodza et al., 2012; Ferreira et al., 2016). Similarly, a number of food- and water-associated *Arcobacter* outbreaks have been described (reviewed by Ferreira et al., 2016). However, arcobacters are not routinely examined for, and their true prevalence remains undetermined. Nonetheless, various studies have shown them to be widely distributed in foods, including shellfish (Levican et al., 2014; Ferreira et al., 2016; Mottola et al., 2016), in which *A. cryaerophilus* has been found in up to 25% of mussels and clams examined (Mottola et al., 2016). Similar studies in India have identified other *Arcobacter* spp. in shellfish but not *A. cryaerophilus* (Laishram et al., 2016; Rathlavath et al., 2017). These studies, together with this report, indicate that the prevalence and distribution of different *Arcobacter* species varies from nation to nation. We note here that our isolation methods were aimed at recovering mainly species implicated as emerging pathogens, and thus the presence of other, environmentally associated species cannot be discounted. However, we can confirm that *A. cryaerophilus* occurs in shellfish from Mediterranean and New Zealand waters.

We believe our study is the first to describe *Arcobacter* spp. in Australasian shellfish and the first to identify a putative virulence plasmid in this group. Previous studies have examined arcobacters of human and animal origin for plasmids; where found, virulence attributes have not been identified (Harrass et al., 1998; Douidah et al., 2014). References validating genes identified on the plasmid described here as virulence determinants are given in Table 1. In wastewater environments, arcobacters have been described as “keystone members …potentially involved in cross-border exchanges between distant Gram-positive and Gram-negative phyla” (Jacquiod et al., 2017). Our isolates were not recovered from areas exposed to wastewater contamination, but this does not preclude the potential for genetic exchange in their natural environments. Various genes identified on the plasmid reported here are involved with genetic movement and integration (Table 1). Given that our understanding of horizontal gene transfer mechanisms is not exhaustive (Toussaint and Chandler, 2012), the potential of intra- and interspecies transference of virulence attributes in food production environments is supported, with implications for food safety and public health. The presence of an acetyltransferase-coding gene associated (albeit not exclusively) with AMR (Vetting et al., 2005) is noteworthy, given the dramatic increase in AMR among many bacterial species, and the role that horizontal gene transfer plays in this process (World Health Organisation [WHO], 2015). The presence of other AMR (and additional pathogenic) traits in our *A. cryaerophilus* genomes (Table 2) may also represent a potential reservoir for wider gene transfer to other microorganisms.

**TABLE 2:** Annotation, predicted functions and distribution among shellfish *A. cryaerophilus* strains of virulence-associated genes.

**Annotation**	**Function**	**Virulence trait**	**Strains^a^**
*flaA*	Flagellin A	Motility and/or adhesion	M830MA
Flagellar assembly protein H	Flagellar assembly protein H	Motility and/or adhesion	G13RTA, M830MA
Flagellar basal body rod modification protein	Flagellar basal body rod modification protein	Motility and/or adhesion	G13RTA, M830MA
Flagellar basal body rod protein FlgG	Flagellar basal body rod protein FlgG	Motility and/or adhesion	G13RTA, M830MA
Flagellar basal body-associated protein FliL	Flagellar basal body-associated protein FliL	Motility and/or adhesion	M830MA
Flagellar biosynthesis protein FliR	Flagellar biosynthesis protein FliR	Motility and/or adhesion	G13RTA, M830MA
Flagellar filament 33 kDa core protein	Flagellar filament 33 kDa core protein	Motility and/or adhesion	G13RTA, G18RTA
Flagellar hook-associated protein FlgL	Flagellar hook-associated protein FlgL	Motility and/or adhesion	G13RTA, M830MA
Flagellar hook-length control protein FliK	Flagellar hook-length control protein FliK	Motility and/or adhesion	G13RTA, M830MA
Flagellar motor switch protein	Flagellar motor switch protein	Motility and/or adhesion	G13RTA, M830MA
Flagellin *N*-methylase	Flagellin *N*-methylase	Motility and/or adhesion	G13RTA, M830MA
*flgB*	Flagellar basal body rod protein FlgB	Motility and/or adhesion	G13RTA, G18RTA, M830MA
*flgC*	Flagellar basal-body rod protein FlgC	Motility and/or adhesion	G13RTA, G18RTA, M830MA
*flgE1*	Flagellar hook protein FlgE	Motility and/or adhesion	G13RTA, G18RTA, M830MA
*flgG*	Flagellar basal-body rod protein FlgG	Motility and/or adhesion	G13RTA, G18RTA, M830MA
*flgH*	Flagellar L-ring protein	Motility and/or adhesion	G13RTA, G18RTA, M830MA
*flgI*	Flagellar P-ring protein	Motility and/or adhesion	G13RTA, G18RTA, M830MA
*flgK*	Flagellar hook-associated protein 1	Motility and/or adhesion	G13RTA, G18RTA, M830MA
*flhA*	Flagellar biosynthesis protein FlhA	Motility and/or adhesion	G13RTA, G18RTA, M830MA
*flhB1*	Flagellar biosynthetic protein FlhB	Motility and/or adhesion	G13RTA, G18RTA, M830MA
*flhF*	Flagellar biosynthesis protein FlhF	Motility and/or adhesion	G13RTA, G18RTA, M830MA
*fliD*	Flagellar hook-associated protein 2	Motility and/or adhesion	G13RTA, G18RTA, M830MA
*fliE*	Flagellar hook-basal body complex protein FliE	Motility and/or adhesion	G13RTA, G18RTA, M830MA
*fliF*	Flagellar M-ring protein	Motility and/or adhesion	G13RTA, G18RTA, M830MA
*fliG*	Flagellar motor switch protein FliG	Motility and/or adhesion	G13RTA, G18RTA, M830MA
*fliI*	Flagellum-specific ATP synthase	Motility and/or adhesion	G13RTA, G18RTA, M830MA
*fliM*	Flagellar motor switch protein FliM	Motility and/or adhesion	G13RTA, G18RTA, M830MA
*fliN1*	Flagellar motor switch protein FliN	Motility and/or adhesion	G13RTA, G18RTA, M830MA
*fliP*	Flagellar biosynthetic protein FliP	Motility and/or adhesion	G13RTA, G18RTA, M830MA
*fliQ*	Flagellar biosynthetic protein FliQ	Motility and/or adhesion	G13RTA, G18RTA, M830MA
*fliS*	Flagellar protein FliS	Motility and/or adhesion	G13RTA, G18RTA, M830MA
*fliW2*	Flagellar assembly factor FliW2	Motility and/or adhesion	G13RTA, G18RTA, M830MA
*hag*	Flagellin	Motility and/or adhesion	G13RTA, G18RTA
*motB*	Motility protein B	Motility and/or adhesion	G18RTA
*ylxH*	Flagellum site-determining protein YlxH	Motility and/or adhesion	G13RTA, G18RTA, M830MA
*acrB*	Multidrug efflux pump subunit AcrB	Antimicrobial resistance	G13RTA, G18RTA, M830MA
*adh2*	Long-chain-alcohol dehydrogenase 2	Antimicrobial resistance	G18RTA
*arnA*	Bifunctional polymyxin resistance protein ArnA	Antimicrobial resistance	G13RTA
*arsB*	Arsenical pump membrane protein	Antimicrobial resistance	G18RTA
*arsC1*	Glutaredoxin arsenate reductase	Antimicrobial resistance	G18RTA
*arsC2*	Arsenate reductase	Antimicrobial resistance	G18RTA
*bcr*	Bicyclomycin resistance protein	Antimicrobial resistance	G13RTA, M830MA
*bepC*	Outer membrane efflux protein BepC	Antimicrobial resistance	G18RTA
*bepD*	Efflux pump periplasmic linker BepD	Antimicrobial resistance	G18RTA
*bepE*	Efflux pump membrane transporter BepE	Antimicrobial resistance	G13RTA, G18RTA, M830MA
*bepF*	Efflux pump periplasmic linker BepF	Antimicrobial resistance	G13RTA, M830MA
Enterobactin exporter EntS	Enterobactin exporter EntS	Antimicrobial resistance	G13RTA
*hcpA*	Beta-lactamase HcpA	Antimicrobial resistance	M830MA
*hcpC*	Putative beta-lactamase HcpC	Antimicrobial resistance	G13RTA, M830MA
*lmrA*	Multidrug resistance ABC transporter ATP-binding and permease protein	Antimicrobial resistance	G13RTA
*marA*	Multiple antibiotic resistance protein MarA	Antimicrobial resistance	M830MA
*mdtB*	Multidrug resistance protein MdtB	Antimicrobial resistance	G13RTA, G18RTA, M830MA
*mexA*	Multidrug resistance protein MexA	Antimicrobial resistance	G13RTA, G18RTA, M830MA
*mexB*	Multidrug resistance protein MexB	Antimicrobial resistance	G13RTA, G18RTA, M830MA
*mrdA*	Penicillin-binding protein 2	Antimicrobial resistance	G13RTA, G18RTA, M830MA
*pbpF*	Penicillin-binding protein 1F	Antimicrobial resistance	G13RTA, G18RTA
Putative multidrug export ATP-binding/permease protein	Putative multidrug export ATP-binding/permease protein	Antimicrobial resistance	G13RTA, G18RTA
*srpC*	Putative chromate transport protein	Antimicrobial resistance	G18RTA
*ttgA*	Putative efflux pump periplasmic linker TtgA	Antimicrobial resistance	G13RTA, G18RTA, M830MA
*ttgC*	Putative efflux pump outer membrane protein TtgC	Antimicrobial resistance	G13RTA, M830MA
*ttgI*	Toluene efflux pump outer membrane protein TtgI	Antimicrobial resistance	G18RTA
*ykkD*	Multidrug resistance protein YkkD	Antimicrobial resistance	G18RTA
*btuB*	Vitamin B12 transporter BtuB	Fe acquisition	G18RTA
*fbpC*	Fe(3+) ions import ATP-binding protein FbpC	Fe acquisition	G13RTA
Ferredoxin–NADP reductase	Ferredoxin–NADP reductase	Fe acquisition	G13RTA
*futA1*	Iron uptake protein A1	Fe acquisition	G13RTA
Gram-negative bacterial TonB protein	Gram-negative bacterial TonB protein	Fe acquisition	M830MA
*hemE*	Uroporphyrinogen decarboxylase	Fe acquisition	G18RTA
*hemH1*	Ferrochelatase	Fe acquisition	G18RTA
*hmuT*	Hemin-binding periplasmic protein HmuT	Fe acquisition	G13RTA, G18RTA, M830MA
*hmuU*	Hemin transport system permease protein HmuU	Fe acquisition	G13RTA, G18RTA, M830MA
*hmuV*	Hemin import ATP-binding protein HmuV	Fe acquisition	G13RTA, G18RTA
*hssS*	Heme sensor protein HssS	Fe acquisition	G13RTA, G18RTA, M830MA
*hxuA*	Heme/hemopexin-binding protein	Fe acquisition	G13RTA, M830MA
*hxuB*	Heme/hemopexin transporter protein	Fe acquisition	G13RTA, M830MA
*isdE*	High-affinity heme uptake system protein IsdE	Fe acquisition	G18RTA
*tdhA*	TonB-dependent heme receptor A	Fe acquisition	G13RTA
*esiB1*	Secretory immunoglobulin A-binding protein EsiB	Immunoavoidance	G18RTA
Plasmid stabilization system protein	Plasmid stabilization system protein	Plasmid stabilization	G13RTA
*virF*	Virulence regulon transcriptional activator VirF	Virulence regulator	G18RTA
*epsF*	Type II secretion system protein F	Toxin secretion	G13RTA
*hxcR*	Putative type II secretion system protein HxcR	Toxin secretion	G13RTA
*prsE*	Type I secretion system membrane fusion protein PrsE	Toxin secretion	G18RTA
Putative two-component membrane permease complex subunit SMU 747c	Putative two-component membrane permease complex subunit SMU_747c	Toxin secretion	G18RTA
*bvgS1*	Virulence sensor protein BvgS	Virulence gene regulation	M830MA
*bvgS2*	Virulence sensor protein BvgS	Virulence gene regulation	M830MA
*bvgS3*	Virulence sensor protein BvgS	Virulence gene regulation	M830MA

*^*a*^Results for strain M830A not shown since genome analysis and isolation history indicated this to represent a clone of M830MA.*

The World Health Organization has emphasized the need for improved understanding of mechanisms of antibiotic resistance appertaining to food and water consumption (World Health Organisation [WHO], 2015). As the evidently first description of a putative virulence plasmid in arcobacters found in shellfish, this study extends our knowledge of potential AMR reservoirs. It is worth noting that our initial observation was made only through complete genome analysis; the use of draft genomes may overlook plasmid carriage, resulting in underreporting of important attributes. Land et al. (2014) determined quality metrics for 32,000 publicly available whole genome sequences, finding some 10% of these were of a questionable standard. Their study found completed genome sequences overwhelmingly attained higher quality scores. Moreover, a subsequent study concluded that sequencing technologies generating shorter sequence reads (i.e., the genome sequence is encompassed in many contiguous fragments) present major difficulties for bioinformatics algorithms seeking to analyze such data (Land et al., 2015). Taken together, it is perhaps not surprising that our study only identified the putative virulence plasmid described here when complementary approaches for generating the complete genome sequence were used. Short-read second generation sequencing remains the most commonly used and cost-effective genome sequencing strategy for bacterial genomes (Land et al., 2015), but as our study indicates, the reduced financial cost can come at a price for biological data that may be of significance.

The pathogenesis of *Arcobacter* infections is poorly understood, despite their long association with human disease (Ferreira et al., 2016). Our *A. cryaerophilus* strains possessed 63–76 genes with known or putative virulence function (Table 2), in addition to those identified on the plasmid. Most functions are conserved between strains and include features for motility and adhesion, heme acquisition, hemolysin or toxin production, and various traits associated with AMR: a feature for which arcobacters are especially noted (On et al., 1996; Ferreira et al., 2016). The importance of this finding is pertinent, given that shellfish are often consumed with minimal treatment.

In summary, we have confirmed for the first time that New Zealand shellfish may harbor emerging pathogenic *Arcobacter* species that have been isolated from cases of human gastroenteritis. Further studies are required to determine more comprehensively the prevalence and distribution of these bacteria for a more complete risk assessment. Of more significance may be the observation that arcobacters may harbor plasmids that contain genes encoding for a variety of virulence and related functions, including those associated with AMR, invasion, immunoavoidance and cytotoxicity. We have determined that the carriage of such plasmids may not always be recognized where only draft (incomplete) genome sequences are determined. Additional studies are needed to assess the wider- and longer-term implications of these results.

## Data Availability

The datasets generated for this study can be found in Genbank, SNQM01000000, SNQL01000000, CP026655, CP026656, and Bankit2207814 M830_plasmid MK715471.

## Author Contributions

SO conceived and coordinated the study and wrote the manuscript. DA isolated the strains described. WM supplied reference whole genome sequences, undertook the phylogenetic analysis, and provided annotation of the plasmid. DL undertook genome annotation and complementary plasmid annotation. SW phenotyped the strains. AM antibiotyped the strains. VC extracted genomic DNA for sequencing and screened isolates for plasmids. GC determined the genome and plasmid sequences for the strains and provided the assemblies.

## Conflict of Interest Statement

The authors declare that the research was conducted in the absence of any commercial or financial relationships that could be construed as a potential conflict of interest.
